# An Improved *Physarum polycephalum* Algorithm for the Shortest Path Problem

**DOI:** 10.1155/2014/487069

**Published:** 2014-03-25

**Authors:** Xiaoge Zhang, Qing Wang, Andrew Adamatzky, Felix T. S. Chan, Sankaran Mahadevan, Yong Deng

**Affiliations:** ^1^School of Computer and Information Science, Southwest University, Chongqing 400715, China; ^2^Unconventional Computing Center, University of the West of England, Bristol BS16 1QY, UK; ^3^Department of Industrial and Systems Engineering, The Hong Kong Polytechnic University, Hung Hum, Kowloon, Hong Kong; ^4^School of Engineering, Vanderbilt University, Nashville, TN 37235, USA

## Abstract

Shortest path is among classical problems of computer science. The problems are solved by hundreds of algorithms, silicon computing architectures and novel substrate, unconventional, computing devices. Acellular slime mould *P. polycephalum* is originally famous as a computing biological substrate due to its alleged ability to approximate shortest path from its inoculation site to a source of nutrients. Several algorithms were designed based on properties of the slime mould. Many of the *Physarum*-inspired algorithms suffer from a low converge speed. To accelerate the search of a solution and reduce a number of iterations we combined an original model of Physarum-inspired path solver with a new a parameter, called energy. We undertook a series of computational experiments on approximating shortest paths in networks with different topologies, and number of nodes varying from 15 to 2000. We found that the improved *Physarum* algorithm matches well with existing Physarum-inspired approaches yet outperforms them in number of iterations executed and a total running time. We also compare our algorithm with other existing algorithms, including the ant colony optimization algorithm and Dijkstra algorithm.

## 1. Introduction

Shortest path problem (SPP) is one of the fundamental problems in the field of network optimization: given a network, it is to find a path between two nodes such that the sum of the weights of its edges is minimized. Due to its wide application in many practical applications, for example transportation of food and commodities [1–4], wireless networks [5, 6], complex networks [7–10], and so forth [11–16]. A number of researchers have developed many efficient algorithms to deal with this problem. For example, one of the most famous algorithms, Dijkstra algorithm [17] was proposed by Edsger Dijkstra in 1959 to solve the single-source shortest path problem. Bellman-Ford algorithm [18] is another well-known algorithm that computes the shortest paths starting from a single source vertex to all of the other vertices in a weighted graph. Also, label correcting algorithm has been proposed by various researchers to deal with this problem [19, 20]. However, these algorithms have one common feature: they need excessive computational time when the scale of the network becomes very large. As a result, many bioinspired algorithms have emerged, such as genetic algorithm [21–23], ant colony algorithm [24], and particle swarm optimization [25].

With regard to future and emergent computing architectures, a first two-dimensional cellular automaton computing shortest path was designed in [26]. The algorithm was used in developing a reaction-diffusion chemical processor to compute a collision-free shortest path in [27]. Recently, an amoeboid organism,* Physarum polycephalum* has been shown to be capable of solving many graph theoretical problems [28–30], including finding the shortest path [30–32], network design [33–39], population migration [40] and others [35, 41–43]. Inspired by this intelligent organism, a path finding mathematical model has been constructed [44]. Moreover, this organism has been shown to be able to form networks with features matching those of motorway networks [38]. In addition, Baumgarten et al. have proved that the mass of mold will eventually converge to the shortest path of the network that the mold lies on [45].

However, when the original* Physarum polycephalum* model is implemented to handle shortest path problem, it needs substantial, often to a degree of excess, number of iterations. In present paper we aimed to improve the efficiency of the original* Physarum polycephalum* model. Here, a new parameter called “energy” is incorporated with the original model. We call the new method as improved* Physarum polycephalum* algorithm (IPPA). In fact, many systems have implied different kinds of “energy” when dealing with the shortest path problem. A number of them employ this kind of “energy” to solve many optimization problems [46–48]. For example, Taherian et al. [49] make full use of the* Resistive Network* concept to simulate the trust networks, in which every node in the trust network is mapped to a node in the resistive network, where the resistors' values are inversely proportional to the trust values.

Fuerstman et al. [50] use the pressure-driven flow in microfluidic network to deal with the maze-like problems by searching all the possible solutions in a parallel way. Liu et al. [51, 52] found that current flows along the branch with lower impedance in circuit are fundamentally similar to the aim of path planning for a shorter path with better feasibility in the map. They employed this phenomenon to find out a short and wide path with light traffic jam for robots, which overcome a lot of shortcomings of the previous approaches. Zelek [53] treats analogous representations of harmonic functions as Markov chains and combines them with resistor networks to develop a novel method to handle the dynamic path planning problem.

Let us incorporate the parameter “energy” with the original* Physarum solver*. Advantages of our approach are manifold. It completes the* Physarum* model with rather physical notion of energy. During the actual expanding process of* Physarum*, it needs to consume energy to expand its tubes. At the same time, its tubes can absorb energy from the surrounding environment. As a result, there is a trade-off between the consumption and the absorption of the energy. By the introduction of the parameter “energy”, it makes this mode more effective when we employ it to design adaptive networks. By binging in this parameter, the executing time and iterations of the original algorithm has been decreased to a great extent. To the authors' knowledge, this is the first attempt to combine “energy” parameter into the* Physarum* model. In addition, we have compared the efficiency of the proposed method and the original (or basic)* Physarum polycephalum* algorithm when they deal with the networks with various network topologies and nodes. Also, we have shown its advantages by comparing with other existing algorithms, including the ant colony optimization algorithm and Dijkstra algorithm.

The remainder of this paper is organized as follows.  Section 2 briefly introduces the mathematical model of* Physarum polycephalum*.  Section 3 presents the improved* Physarum polycephalum* algorithm for path finding. We compare the improved* Physarum polycephalum* algorithm with the basic* Physarum polycephalum* algorithm and other existing algorithms in Section 4.  Section 5 concludes this paper.

## 2. *Physarum polycephalum* Inspired Shortest Path Finding Model

*Physarum polycephalum* is a large, single-celled amoeboid organism forming a dynamic tubular network connecting the discovered food sources during foraging. The mechanism of tube formation can be described as tubes become thicker in a given direction when shuttle streaming of the protoplasm persists in that direction for a certain time. It implies positive feedback between flux and tube thickness, as the conductance of the sol is greater in a thicker channel. With this mechanism, a mathematical model illustrating the shortest path finding has been constructed [44].

Suppose that the shape of the network formed by the* Physarum* is represented by a graph, in which a plasmodial tube refers to an edge of the graph, and a junction between tubes refers to a node. Two special nodes labeled as *N*_1_ and *N*_2_ act as the starting node and ending node, respectively. The other nodes are labeled as *N*_3_, *N*_4_, *N*_5_, *N*_6_, and so forth. The edge between node *N*_*i*_ and *N*_*j*_ is expressed as *M*_*ij*_. The parameter *Q*_*ij*_ denotes the flux through tube *M*_*ij*_ from node *N*_*i*_ to *N*_*j*_. Assume the flow along the tube as an approximately Poiseuille flow; the flux *Q*_*ij*_ can be expressed as

(1) $$\begin{array}{c} Q_{ij}=\frac{D_{ij}}{L_{ij}}\left(p_{i}-p_{j}\right), \end{array}$$

where *p*_*i*_ is the pressure at the node *N*_*i*_; *D*_*ij*_ is the conductivity of the tube *M*_*ij*_; *L*_*ij*_ is its length.

By considering that the inflow and outflow must be balanced, we have:

(2) $$\begin{array}{c} \underset{j\neq 1,2}{\sum }Q_{ij}=0. \end{array}$$

For the source node *N*_1_ and the sink node *N*_2_ the following two equations hold:

(3) $$\begin{array}{c} \underset{i}{\sum }Q_{i1}+I_{0}=0,\\ \underset{i}{\sum }Q_{i2}-I_{0}=0, \end{array}$$

where *I*_0_ is the flux flowing from the source node, and *I*_0_ is a constant value here.

In order to describe such an adaptation of tubular thickness we assume that the conductivity *D*_*ij*_ changes over time according to the flux *Q*_*ij*_. The following equation for the evolution of *D*_*ij*_(*t*) can be used:

(4) $$\begin{array}{c} \frac{d}{dt}D_{ij}=f\left(\left|Q_{ij}\right|\right)-rD_{ij}, \end{array}$$

where *r* is a decay rate of the tube. It can be obtained that the equation implies that the conductivity ends to vanish if there is no flux along the edge, while it is enhanced by the flux. The *f* is monotonically increasing continuous function satisfying *f*(0) = 0.

Then the network Poisson equation for the pressure can be obtained from (1)to(3) as follows:

(5) $$\begin{array}{c} \underset{i}{\sum }\frac{D_{ij}}{L_{ij}}\left(p_{i}-p_{j}\right)=\{\begin{array}{cc} +1 & \text{for}\;j=1,\\ -1 & \text{for}\;j=2,\\ 0 & \text{otherwise}. \end{array} \end{array}$$

By setting *p*_2_ = 0 as a basic pressure level, all *p*_*i*_ can be determined by solving (5) and *Q*_*ij*_ can also be obtained.

In this paper, *f*(*Q*) = |*Q*| is used. With the flux calculated, the conductivity can be derived, where (6) is used instead of (4), adopting the functional form *f*(*Q*) = |*Q*|:

(6) $$\begin{array}{c} \frac{D_{ij}^{n+1}-D_{ij}^{n}}{\delta t}=\left|Q\right|-D_{ij}^{n+1}. \end{array}$$

In order to illustrate the basic process of* Physarum polycephalum* algorithm, one simple example is shown.

**Example**

**Figure 1:**
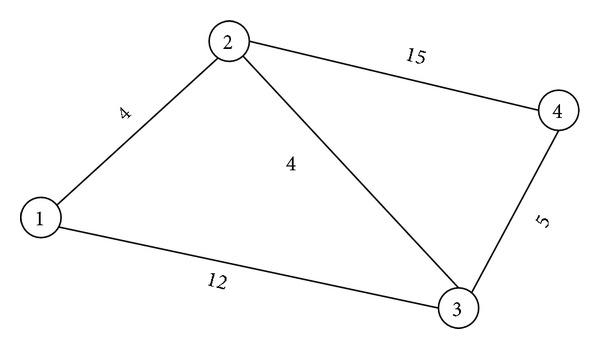
A small network with four nodes.

**Figure 2:**
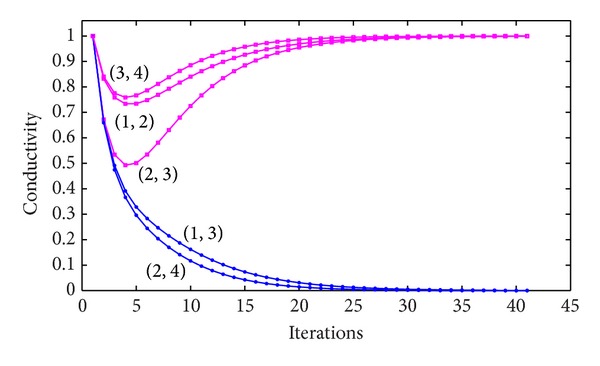
The changing trend of the conductivity associated with every edge.

Consider the network shown in Figure 1; as can be seen, the numbers along each edge represent the edge length. Now, the shortest path between node 1 and node 4 needs to be found. First of all, we initialize the conductivity of all edges as 1. By implementing our proposed method, the conductivity associated with each edge is recorded, which is shown in Figure 2.

As can be seen in Figure 2, the flux of edges (1,2), (2,3),and (3,4) converge to 1 while that of the other edges converge to 0. Therefore, the shortest path found by the proposed method is 1 → 2 → 3 → 4 and the results are the same as that of other algorithms, such as Dijkstra algorithm.

## 3. Improved* Physarum polycephalum* Algorithm

In this section, the improved* Physarum polycephalum* algorithm is introduced in detail. In this approach, a new parameter called “energy” is incorporated with the original mathematical model. The maintenance of the tubes in* Physarum polycephalum* model needs to consume energy while this energy comes from the flowing nutrients in the tubes. When the obtained energy is greater than the consumed energy, these tubes become coarser and the conductivity increases. Otherwise these tubes will vanish. The change of the tubes results in the change of the allocated flux associated with each tube. In turn, the variance of the flux changes the energy balance in the tubes. As a consequence, the tube changes further. Through a series of change associated with energy, flux, and tube, the* Physarum polycephalum* tends to converge to a steady state.

First of all, the energy *E*, the flux *Q*, and the conductivity *D* are defined as below

(7a) $$\begin{array}{c} E_{1}=f\left(Q\right), \end{array}$$

(7b) $$\begin{array}{c} E_{2}=g\left(D\right), \end{array}$$

(7c) $$\begin{array}{c} \Delta D=h\left(E_{3}\right), \end{array}$$

where (7a) represents how much energy can be provided by the tube when its flux reaches *Q*; (7b) denotes the energy consumed by the tube with conductivity equal to *D*; (7c) means how the conductivity will change when the remaining energy (here, remaining energy is equal to the energy provided by the flux minus the energy consumed by the tube itself) is *E*_3_.

Therefore, (6) is changed to the following form:

(8) $$\begin{array}{c} \Delta D_{ij}=h\left(f\left(Q_{ij}-g\left(D_{ij}\right)\right)\right)\times \Delta t. \end{array}$$

In the differential form, it will be

(9) $$\begin{array}{c} \frac{dD_{ij}}{dt}=h\left(f\left(Q_{ij}-g\left(D_{ij}\right)\right)\right), \end{array}$$

which is similar to (6). But it is in more accordance with biological significance.

In what follows, we will construct the functions *f*, *g*, and *h* in (7a)–(7c). Equation (7a) reflects the relationship between the flux and the energy. In the original* Physarum polycephalum* model, this is defined by the absolute value of *Q* as shown in (6). However, it breaks the basic principle of conservation of energy. Consider a period of tube containing *Q*'s flux; then the energy acquired by this tube is *E* = |*Q*| according to (6). If we regard the above tube as two connected tubes, calculate their energy, respectively; it is found that they all get the energy |*Q*|. Therefore, the flux *Q* offers 2|*Q*|'s energy for this tube. Obviously, this fact contradicts with the previous result. It means that the relationship between the flux and the energy described by (6) in Section 2 is not reasonable.

Here, in order to satisfy the law of conservation of energy, we assume that the total energy provided by the flux beginning from the starting node to the ending node is constant and has nothing to do with the path. Therefore, the function *f* is defined as follows:

(10) $$\begin{array}{c} f\left(Q_{ij}\right)=\frac{Q_{ij}\times \left(p_{i}-p_{j}\right)}{p_{s}-p_{e}}, \end{array}$$

where *s* and *e* represent the starting node and the ending node, respectively, *p*_*i*_ and *p*_*j*_ denote the pressure at the node *i* and node *j*.

Consider (7b); it reflects the consumed energy for maintaining the tubes. Naturally, the consumed energy is not only relevant with the conductivity, but also with the length of the tubes. Thus, we define it as follows:

(11) $$\begin{array}{c} g\left(D_{ij}\right)=D_{ij}\times L_{ij}. \end{array}$$

Similarly, the effect of the energy on the change of the conductivity is also relevant with the length of the tube. The longer the tube is, the more energy it consumes. As a result, we define (7c) as

(12) $$\begin{array}{c} h\left(E_{3}\right)=\frac{E_{3}}{L_{ij}}. \end{array}$$

After we combine (10)–(12), the following equation can be constructed:

(13) $$\begin{array}{c} \frac{dD_{ij}}{dt}=\frac{Q_{ij}\times \left(p_{i}-p_{j}\right)}{L_{ij}\times \left(p_{s}-p_{e}\right)}-D_{ij}. \end{array}$$

Based on the above constructed model, the main procedures of this model for the shortest path problem are presented as Algorithm 1.

There are several possible solutions to decide when to stop execution of Algorithm 1, such as the maximum number of iterations is arrived, the flux through each tube remains unchanged.

As for the time complexity of this bioinspired algorithm, it is *o*(*n* ^3^), where *n* is the number of the nodes in the network. When we implement this algorithm, it is necessary to solve the linear equations shown in (5). Although the time complexity is substantial; different strategies can be applied to reduce the time cost, such as parallel computing and approximate approaches to solve the equations, and these advantages make this algorithm promising.

## 4. Comparison of Algorithms

In order to demonstrate the efficiency of the proposed method, a number of experiments have been conducted on different datasets. In addition, we have compared the computational results between our method and the existing path finding mathematical model. In addition, we have compared the computational efficiency with other state-of-the-art algorithms, including Dijkstra algorithm and Ant Colony Optimization Algorithm. All the approaches are tested on networks with random and varying topologies through computer simulations using Matlab on an Intel Pentium Dual-Core E5700 processor (3.00 GHz) with 2 GB of RAM under Windows Seven.

As for the random networks, they are generated by* erdos.renyi.game* function of the igraph package in R language [54].  Table 1 shows the size of testing networks. In this paper, we test the proposed method on 15 networks with different topologies and their network size ranges from 15 to 2000. Each instance is run for 40 times, and we compute the average executing time and average accuracy.

### 4.1. Comparison with the Basic* Physarum polycephalum* Algorithm

In order to ensure that there exists at least one path from starting node to ending node in the network, we make the network fully connected. The length of each edge is uniformly distributed integer ranging from 1 to 100. The scale of the tested network varies from 15 to 2000. In our experiments, when ∑_*i*,*j*=1,2,…,*n*_(|*D*_*ij*_^count+1^ − *D*_*ij*_^count^|) ≤ 0.01 (*D*_*ij*_^count+1^  and  *D*_*ij*_^count^ refer to the conductivity associated with the edge *L*_*ij*_ during the *n* + 1th and *n*th iteration, resp., and *n* represents the scale of the network), the procedure ends.

**Figure 3:**
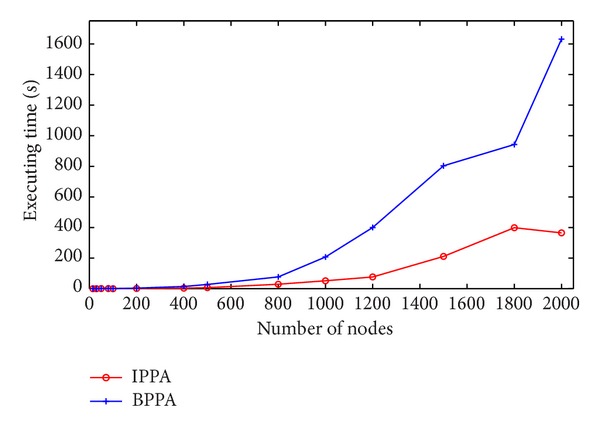
Comparison of executing time on randomly generated networks. IPPA refers to the improved* Physarum polycephalum* algorithm while BPPA represents the basic* Physarum polycephalum* algorithm.

**Figure 4:**
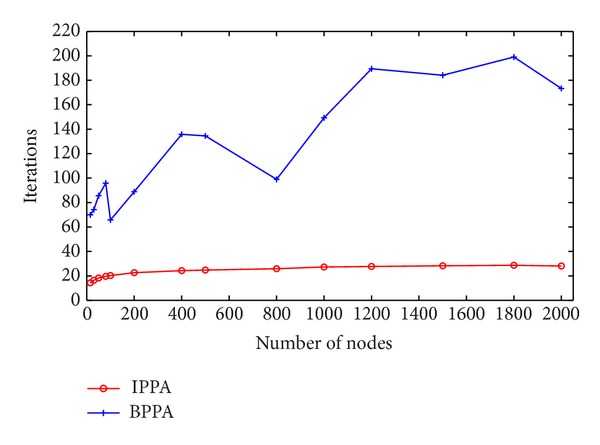
Comparison of running iterations on randomly generated networks. IPPA refers to the improved* Physarum polycephalum* algorithm while BPPA represents the basic* Physarum polycephalum* algorithm.

Normally, the performance of an algorithm is its accuracy and executing time. First of all, consider the accuracy; both IPPA (short for improved* Physarum polycephalum* algorithm) and BPPA (short for basic* Physarum polycephalum* algorithm) are capable of finding the optimal path as Dijkstra algorithm with one hundred percent. Secondly, as for running time, the results are summarized in Figures 3 and 4. It is obvious that IPPA outperforms BPPA at all testing instances on both executing time and running iterations. Moreover, when the scale of the network gradually increases, the advantage of IPPA becomes more noticeable. From the view of running iterations as shown in Figure 4, due to the randomness of the edge length, for both IPPA and BPPA, executing iterations fluctuate slightly. However, as can be seen, IPPA still has obvious priority when compared with BPPA for all the testing instances. In addition, IPPA is more stable than BPPA. In summary, the above features make IPPA more applicable to real-world applications.

### 4.2. Comparison with Other State-of-the-Art Algorithms

Here, we compare the improved* Physarum polycephalum* algorithm with the classical Dijkstra algorithm [17] and Ant Colony Optimization (ACO) algorithm [55]. We focus on two important factors: a time of execution and a degree of accuracy.

In all experiments discussed here the numeric parameters, except when explicitly shown, are set to the following values: *α* = 2, and it denotes the preference weight of pheromone trail; *β* = 5, and it represents the preference weight of the heuristic parameter *η*, and *η* is the inverse of the distance between the nodes. The global update evaporation rate *ρ* is equal to 0.1. Both the size of the ants in each ant colony system and the maximum number of iterations are equal to the number of nodes in each network.

**Figure 5:**
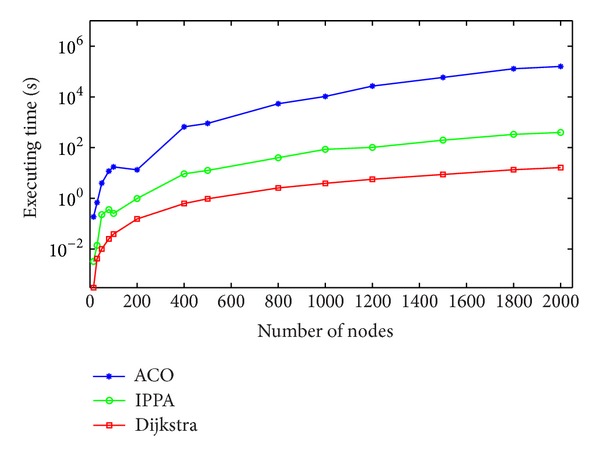
Comparison of running time on randomly generated networks. ACO refers to the ant colony optimization algorithm while IPPA refers to the improved* Physarum polycephalum* algorithm.

**Figure 6:**
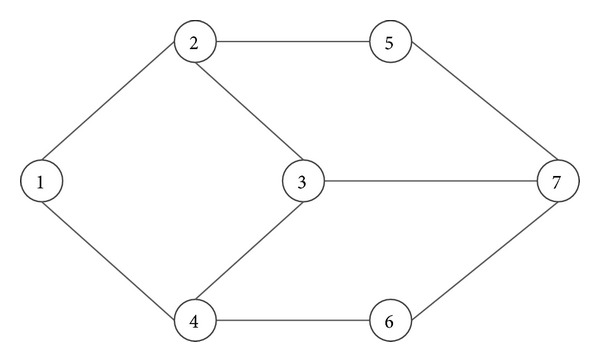
A simple network with 7 nodes.

**Figure 7:**
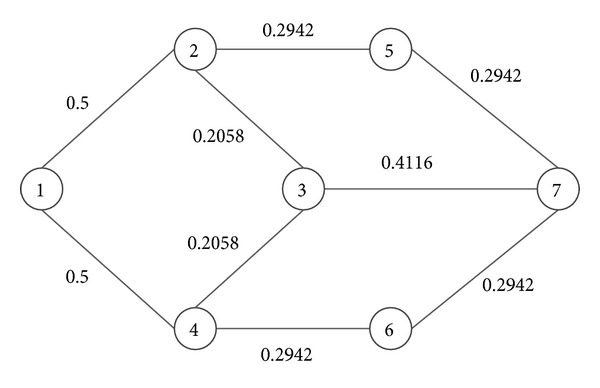
The flux associated with each edge in the* Physarum polycephalum* algorithm.

As can be seen in Figure 5, Dijkstra algorithm is faster than the improved* Physarum polycephalum* algorithm. At the same time, the improved* Physarum polycephalum* algorithm is faster than the ant colony optimization algorithm. It is obvious that the improved* Physarum polycephalum* algorithm outperforms the ant colony optimization algorithm when dealing with the shortest path problem. As for the Dijkstra algorithm, although it is faster than the improved* Physarum polycephalum* algorithm, it needs extra operation before it can be implemented directly to solve the shortest path problem when there is more than one shortest path in the network. For instance, for the network shown in Figure 6, the length of all the edges is  1. If we want to find the shortest path between node  1 and node  7, it will be hard for the classical Dijkstra algorithm to solve this problem. On the contrary, it is very simple for the improved* Physarum polycephalum* algorithm. As can be seen in Figure 7, each edge is associated with the flux. If we want to find all the shortest paths from node  1 to node  7, we can follow the direction of the flow to construct these paths, which is very easy to realize.

**Figure 8:**
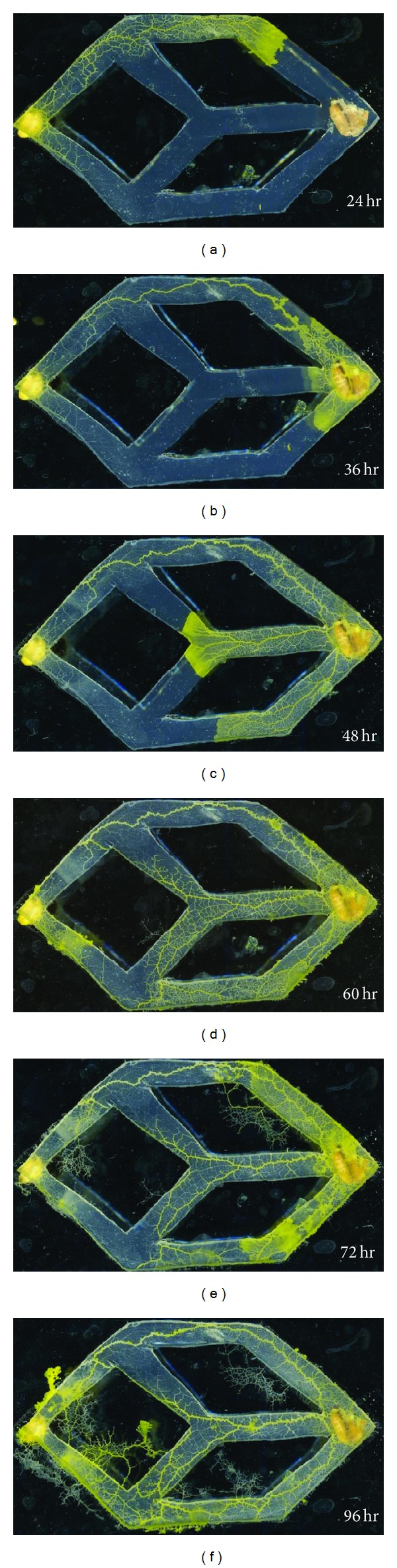
The process of shortest path formulation by real* Physarum polycephalum*. From (a) to (f) the* Physarum polycephalum* grew out of the source node and gradually constructed the shortest path from the source node to the ending node.

In order to prove this point, we have observed how* Physarum* will behave in this network and compare the result with that shown in Figure 7. As can be seen in Figure 8, it has shown the specific process of* Physarum* connecting the source node and the ending node. Each junction represents the node in the network shown in Figure 6, and* Physarum* is placed in the left side of the container. The* Physarum polycephalum* is allowed to spread along each edge in the network. With the time going, an alternative shortest path is gradually constructed as shown in Figure 8(a). At this stage, the* Physarum polycephalum* does not stop. On the contrary, it starts propagating back to the source as shown in Figures 8(b), 8(c), 8(d), and 8(e). At *t* = 72, the tubes of* Physarum* were connected with the initial food sources. Finally, the* Physarum* yields the network starting from the source node to the ending node shown in Figure 8(f). It can be seen that the real* Physarum* yields similar results as we discuss before. This is a unique feature for* Physarum*. It can retain all the shortest paths in a network, and its process is continuous, which is totally different from Dijkstra algorithm. This is also why the* Physarum* can construct the robust network.

**Figure 9:**
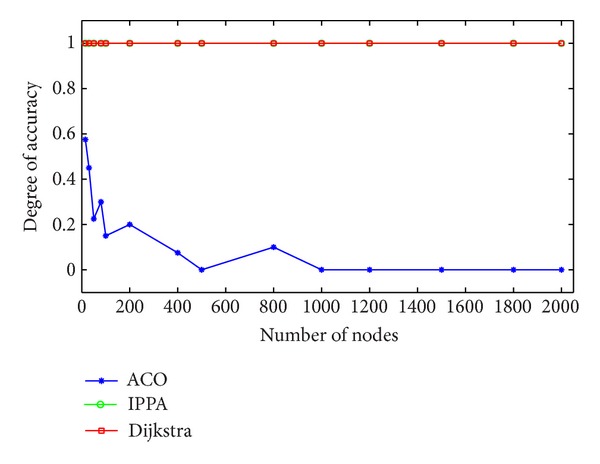
Comparison of accuracy on different algorithms solving the shortest path problem on randomly generated networks. ACO refers to the ant colony optimization algorithm while IPPA refers to the improved* Physarum polycephalum* algorithm.

As for the accuracy of these algorithms, we also have compared it with the ant colony optimization and Dijkstra algorithm. As shown in Figure 9, both the improved* Physarum polycephalum* algorithm and Dijkstra algorithm can find the optimal paths with one hundred percent. However, for the ant colony optimization algorithm, its accuracy decreases gradually with the increase of the network. When compared with the improved* Physarum polycephalum* algorithm and Dijkstra algorithm, its accuracy is very low.

## 5. Conclusion

In this paper, the basic model of* Physarum polycephalum* is combined with a new parameter “energy” to solve the shortest path problem. Through this novel parameter, we make the* Physarum polycephalum* model more reasonable. Furthermore, this parameter helps to accelerate the search speed and to reduce the number of iterations of the basic* Physarum polycephalum* algorithm. The performance of this novel approach is tested on various networks with different structures, and nodes ranging from 15 to 2000. In addition, we compare the proposed method with the original* Physarum polycephalum* model, the ant colony optimization algorithm, and Dijkstra algorithm. The results show that the proposed method outperforms the basic model of* Physarum polycephalum* algorithm and the ant colony optimization algorithm on both running time and executing iterations. Also, when compared with Dijkstra algorithm, it has some obvious advantages, such as finding more than one shortest path at the same time. In the future, we will investigate how to accelerate the speed of solving the linear equations shown in equation (5).

## Figures and Tables

**Algorithm 1:**
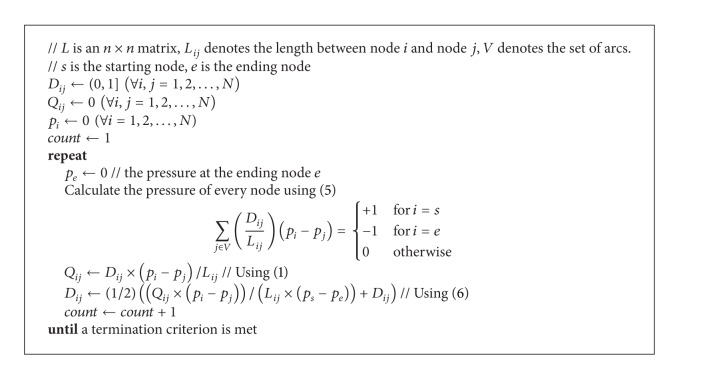
Improved *Physarum polycephalum* algorithm (*L*, *s*, *e*).

**Table 1:** The related parameters of the *erdos.renyi.game* function. In this function, the parameter *P* denotes the probability of drawing an edge between two arbitrary vertices.

Test problem	Number of nodes	Number of edges	*P*
1	15	23	0.20
2	30	45	0.15
3	50	107	0.10
4	80	240	0.08
5	100	304	0.06
6	200	819	0.04
7	250	1124	0.04
8	400	1634	0.02
9	500	2496	0.02
10	800	3229	0.01
11	1000	3950	0.008
12	1200	4347	0.006
13	1500	6822	0.006
14	1800	6521	0.004
15	2000	4044	0.002
